# Mutual influence of secondary and key drug‐resistance mutations on catalytic properties and thermal stability of TEM‐type β‐lactamases

**DOI:** 10.1002/2211-5463.12352

**Published:** 2017-12-11

**Authors:** Vitaly Grigorenko, Igor Uporov, Maya Rubtsova, Irina Andreeva, Dmitrii Shcherbinin, Alexander Veselovsky, Oksana Serova, Maria Ulyashova, Igor Ishtubaev, Alexey Egorov

**Affiliations:** ^1^ Chemistry Faculty M.V. Lomonosov Moscow State University Russia; ^2^ Institute of Biomedical Chemistry Moscow Russia

**Keywords:** antibiotic resistance, drug resistance mutations, molecular dynamics, recombinant TEM‐type β‐lactamases

## Abstract

Highly mutable β‐lactamases are responsible for the ability of Gram‐negative bacteria to resist β‐lactam antibiotics. Using site‐directed mutagenesis technique, we have produced *in vitro* a number of recombinant analogs of naturally occurring TEM‐type β‐lactamases, bearing the secondary substitution Q39K and key mutations related to the extended‐spectrum (E104K, R164S) and inhibitor‐resistant (M69V) β‐lactamases. The mutation Q39K alone was found to be neutral and hardly affected the catalytic properties of β‐lactamases. However, in combination with the key mutations, this substitution resulted in decreased *K*_M_ values towards hydrolysis of a chromogenic substrate, CENTA. The ability of enzymes to restore catalytic activity after exposure to elevated temperature has been examined. All double and triple mutants of β‐lactamase TEM‐1 bearing the Q39K substitution showed lower thermal stability compared with the enzyme with Q39 intact. A sharp decrease in the stability was observed when Q39K was combined with E104K and M69V. The key R164S substitution demonstrated unusual ability to resist thermal inactivation. Computer analysis of the structure and molecular dynamics of β‐lactamase TEM‐1 revealed a network of hydrogen bonds from the residues Q39 and K32, related to the N‐terminal α‐helix, towards the residues R244 and G236, located in the vicinity of the enzyme's catalytic site. Replacement of Q39 by lysine in combination with the key drug resistance mutations may be responsible for loss of protein thermal stability and elevated mobility of its secondary structure elements. This effect on the activity of β‐lactamases can be used as a new potential target for inhibiting the enzyme.

**Abbreviations**
ESBL: extended spectrum β‐lactamases
IR: inhibitor resistant β‐lactamases
MD: molecular dynamics
RMSD: root mean square deviation

Bacterial β‐lactamases determine the main mechanism of resistance to β‐lactam antibiotics, the most widely used class of antibacterial drugs 1, 2, 3. The appearance and spread of these enzymes represent a constant challenge for the clinical treatment of infections and design of new antibiotics and inhibitors. The high mutability of these enzymes indicates mechanisms of genetic adaptation during the course of evolution 4, 5. Elucidation of the role of individual mutations and their mutual effects on enzyme properties is of great interest for understanding the mechanisms of emergence and dissemination of resistance.

TEM‐type β‐lactamases belong to the enzymes of molecular class A, most prevalent in Gram‐negative bacteria 6, 7. At present, 205 unique TEM‐type enzymes are described in the database of β‐lactamases (http://www.lahey.org/Studies/temtable.asp/). These enzymes have a sequence of 286 amino acids. All TEM‐type β‐lactamases represent mutants of β‐lactamase TEM‐1 that differ from it by several single amino acid substitutions (one to seven). Currently, the mutations are found in 89 positions, five of them located in the signal sequence. Evolution of TEM‐type β‐lactamases and the role of individual mutations are actively studied 7, 8, 9, 10, 11, 12. Loops were shown to be the main evolvable regions; they contain 85% of the substitutions with the exception of position 276, which is in the middle of the C‐terminal helix 13. To date, a functional role has been established for no more than 10–12% of mutations. They are divided into two groups: the key mutations and the secondary ones. Key mutations are divided into two types: mutations of residues 104, 164, 238 and 240, expanding the substrate specificity towards cephalosporins of generations III and IV and thus called extended spectrum β‐lactamases (ESBL; phenotype 2be), and mutations of residues 69, 130, 244, 275 and 276, which are responsible for inhibitor‐resistant (IR; phenotype 2br) forms of the enzymes. The key mutations are located close to the active center, while most of the secondary mutations are distant from it, and some of them are surface located. Figure 1 shows the prevalence of various mutated positions among the subtypes of TEM β‐lactamases. The most common are all the key replacements and some of the secondary ones, which include 39, 182 and 265.

**Figure 1:**
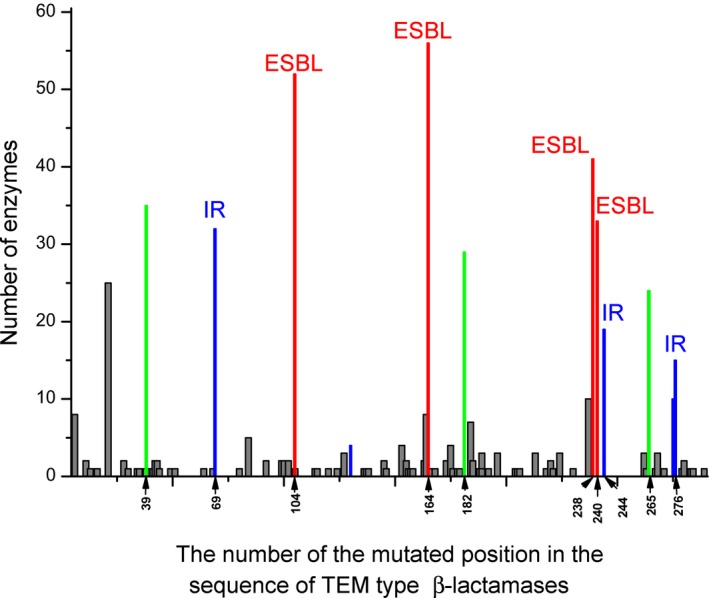
Mutated positions in the sequence of TEM‐type β‐lactamases and the number of enzymes that have substitutions in these positions. Occurrence of mutating residues responsible for the extended spectrum β‐lactamases (ESBL) is marked in red, occurrence of mutating residues responsible for inhibitor‐resistant (IR) enzymes is marked in blue, and occurrence of the most common secondary mutations is marked in green.

The variety of TEM‐type β‐lactamases promotes the study of structure–function relationships. Interaction networks were created for understanding the evolutionary patterns of resistance towards β‐lactams, involving all natural and artificial mutations described 14, 15. Investigation of individual secondary mutations and their relationship to the key drug resistance mutations is also of great interest. Moreover, the stabilizing effect of the secondary mutation M182T in combination with the key mutations G238S and M69I was established and attracted considerable attention 16, 17. Earlier we investigated the role of two secondary mutations (Q39K, M182T) in combination with two key mutations on the stability of β‐lactamase TEM‐72 by means of molecular dynamics (MD) simulation 18. The computational data indicated that these two mutations exhibited opposite effects on the protein structure: the mutation M182T stabilizes while the mutation Q39K might destabilize the protein and increase conformational mobility of β‐lactamase.

The aim of this work was to carry out experimental and structural studies to clarify the role of the mutation Q39K and its combinations with the key drug resistance mutations on the activity and stability of the enzyme. The Q39K mutation was the first natural mutation found in β‐lactamases 19. It is the most common among secondary mutations in TEM‐type β‐lactamases and it was demonstrated to be present in all phenotypes of β‐lactamases. We studied mutant forms of β‐lactamase TEM‐1 having naturally occurring combinations of Q39K with E104K, R164S and M69V as a part of single, double and triple mutants (Fig. 2). The effect of the Q39K mutation in combination with the key mutations consisted of a decrease in *K*_M_ values towards the substrate CENTA and an increase in the thermal inactivation rate. Analysis of the tertiary structure of β‐lactamase TEM‐1 and its mutants performed by computer and MD simulations revealed an increase in the mobility of the site associated with the destruction of a hydrogen bond network.

**Figure 2:**
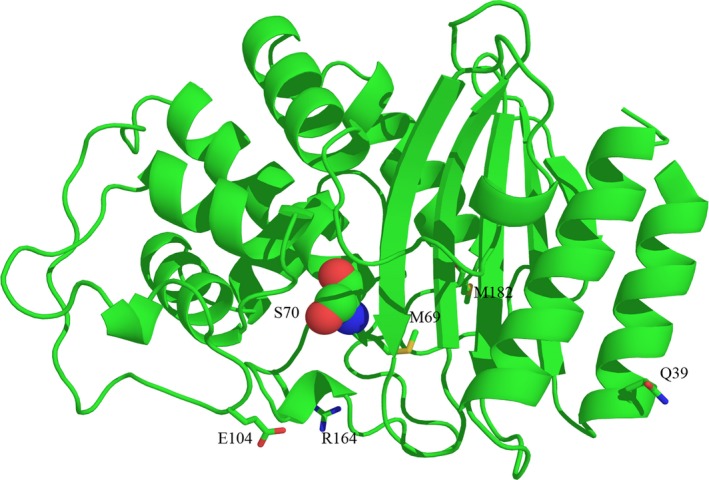
Location of the residues studied in the work on tertiary structure of β‐lactamase TEM‐1 (PDB code 1BTL) in ribbon presentation. Catalytic Ser70 side chain is shown in sphere form and residues Q39, M69, E104, R164 and M182 are shown as sticks. All structural depictions were prepared with pymol.

## Experimental procedures

Reagents were purchased from Sigma‐Aldrich (St Louis, MO, USA) and BD Biosciences (San Jose, CA, USA) and used without further purification. Enzymes for DNA amplification, restriction and modification were purchased from Agilent Technologies (Santa Clara, CA, USA), New England Biolabs (Ipswich, MA, USA) and Thermo Fisher Scientific (Waltham, MA, USA). Oligonucleotides for sequencing and PCR were purchased from Syntol (Moscow, Russia). The plasmid vectors pET‐20 and pET‐24a(+) and *E. coli* strains DH5α and BL21(DE3) were purchased from Merck (Darmshtadt, Germany). The preparative work with DNA was performed using a Qiagen QIAprep Spin Miniprep Kit and a Qiagen QIAquick Gel Extraction Kit (Hilden, Germany). Protein electrophoresis (SDS/PAGE) was performed according to Laemmli 20, using a Thermo Fisher Scientific low molecular mass protein kit (SM0431) for the molecular mass standards. Protein concentration was measured with a Sigma‐Aldrich BCA test kit.

### DNA manipulation and generation of β‐lactamase expression strain

DNA techniques, such as plasmid isolation, transformation of *E. coli*, ligations and restriction analysis were standard methods 21. *E. coli* strain DH5α was used for cloning and BL21(DE3) for protein expression. The cells were cultivated in lysogeny broth (LB) medium (0.5% yeast extract, 1% peptone, 0.5% sodium chloride and 1.5% Bacto agar for solid medium) supplemented with 50 mg·L^−1^ of kanamycin.

### Introduction of mutations by site‐directed mutagenesis

PCR amplification was performed with Pfu DNA polymerase by site‐directed mutagenesis 22 in a total volume of 25 μL in a DNA amplificator (Mastercycler gradient, Eppendorf, Hamburg, Germany) using the following protocol: initial denaturation at 95 °C, 2 min; amplification, 15 cycles of denaturation at 95 °C, 30 s; primer annealing at 55 °C, 1 min; elongation at 72 °C, 7 min; ultimate elongation at 72 °C, 10 min; and cooling the mixture to 4 °C. The restriction enzyme DpnI was added into the PCR mixture obtained and after incubation at 37 °C for 1 h it was used to transform *E. coli* DH5α cells. The previously developed pET‐bla expression vector 23 was used as a template. Expression vector pET‐bla encodes the full length β‐lactamase gene including the signal sequence without any tags. Special primer pairs (Table 1) were used to generate a set of recombinant TEM‐type β‐lactamases.

**Table 1:** Primer pairs for the introduction of single mutations into gene of β‐lactamase TEM‐1 using site‐directed mutagenesis

Mutation in β‐lactamase	Primer direction	Sequence (5′→3′)	Introduced mutation
Q39K	Forward	GATGCTGAAGATAAGTTGGGTGCAC	Q_CAG_39K_AAG_
	Reverse	GTGCACCCAACTTATCTTCAGCATC	
E104K	Forward	GAATGACTTGGTTAAGTACTCACCAG	E_GAG_104K_AAG_
	Reverse	CTGGTGAGTACTTAACCAAGTCATTC	
R164S	Forward	ACTCGCCTTGATAGTTGGGAACCG	R_CGT_164S_AGT_
	Reverse	CGGTTCCCAACTATCAAGGCGAGT	
M69V	Forward	CGTTTTCCAATGGTGAGCACTTTTA	M_ATG_69V_GTG_
	Reverse	TAAAAGTGCTCACCATTGGAAAACG	
M182T	Forward	GTGACACCACGACGCCTGCAGCAATG	M_ATG_182T_ACG_
	Reverse	CATTGCTGCAGGCGTCGTGGTGTCAC	

Underlining signifies a triplet which codes an amino acid.

### Expression and purification of the recombinant TEM‐type β‐lactamases

*E. coli* cells were grown in medium containing 1% Bacto Tryptone (BD Biosciences), 0.5% yeast extract, 1% NaCl, 0.1% glucose and 50 μg·mL^−1^ kanamycin. IPTG was used as an inducer. Cells were grown at 30 °C with stirring (180 r.p.m.) to a value of 0.8–1.2 absorbance units at 600 nm; then they were induced with 0.1 mm IPTG and the cultivation was continued for 5 h. The cells were centrifuged at 3000 ***g*** and 4 °C and stored at −20 °C.

### Isolation and purification of the recombinant β‐lactamases

The periplasmic protein fraction was isolated using osmotic shock. In brief, the cells were thawed on ice, resuspended in a buffer containing 20% sucrose, 1 mm EDTA and 10 mm Tris/HCL, pH 8.0, and incubated on ice for 15 min. The resulting spheroplasts were removed by centrifugation at 10 000 ***g*** at 4 °C for 15 min. The supernatant was dialyzed against 10 mm Tris/HCL (pH 8.0) and used for further purification. Ion exchange chromatography was performed on a SOURCE™ 15Q column (10 cm × 0.75 cm^2^, GE Healthcare Life Sciences, Freiburg, Germany), equilibrated with the same buffer; the recombinant enzyme preparation was eluted with a linear gradient (0–300 mm NaCL, 2 mL·min^−1^). The estimated purity of all the recombinant proteins was of more than 95%. Fractions containing the active enzyme were stored at +4 °C or frozen at −20 °C.

### Determination of kinetic parameters

Activity of β‐lactamases was measured against a chromogenic substrate, CENTA (BD Biosciences, La Jolla, CA, USA) 24 in 50 mm sodium phosphate buffer, pH 7.0 at 25 °C using a Shimadzu UV‐1602 spectrophotometer. CENTA stock solution of 4 mm was prepared in 50 mm sodium phosphate buffer, pH 7.0. The hydrolysis of CENTA was monitored by continuous recording of the absorbance at 405 nm (ε_405_ = 6400 m ^−1^·cm^−1^). The concentration of β‐lactamase in the assay was 0.01 μm. The reaction was initiated by adding CENTA at concentrations of 20, 50, 100 and 200 μm to the solution containing the enzyme. The measurements were made in triplicate. Apparent Michaelis constants (*K*_M_) and *k*_cat_ were determined using a weighted Lineweaver–Burk linearization. The weights were taken as *V*_0_^4^/σ^2^(*V*_0_).

### Study of thermal inactivation

Study of thermal inactivation was carried out at 60 °C in 50 mm phosphate buffer, pH 7.0 with the enzyme concentration 0.1 mg·mL^−1^. The enzyme aliquots withdrawn at time intervals (10, 20, 30, 60, 90, 120 and 180 min) were brought to room temperature, and their residual catalytic activity was measured.

### Molecular dynamics simulations

The spatial structures of β‐lactamases TEM‐1 (PDB code 1BTL) and TEM‐72 (PDB code 3P98) were taken as the enzymes’ initial structures 25, 26. These structures were protonated using the sybyl 8.1 package (Tripos, St Louis, MO, USA). The protein molecule was positioned in a cell filled with TIP3P water molecules and periodic boundary conditions were used during simulations. The total charge of the system was neutralized by adding Na^+^ ions. MD simulations were carried out with the gromacs‐5.1 package (http://www.gromacs.org) using amber99‐SB forcefield. The potential energy of the full system was minimized (150 000 steps), followed by gradual heating up to 300 K (NVT ensemble) and raising pressure (NPT ensemble). Total length of MD trajectories was 25 ns (2 fs time step). Protein structures remained stable in the course of the MD simulation. The conformations saved each 200 ps were analyzed to calculate the number of established intramolecular hydrogen bonds using the gmx_hbond utility from the gromacs‐5.1 package.

## Results

### Production and kinetic study of the recombinant TEM‐type β‐lactamases

To study the role of the Q39K substitution and its combinations with the key drug resistance mutations E104K, R164S and M69V on the activity and stability of TEM‐type β‐lactamases, we produced *in vitro* a number of recombinant TEM‐type β‐lactamases, listed in Table 2. All of them represent the analogs of natural forms of β‐lactamases corresponding to various phenotypes. The recombinant proteins were obtained by a method combining periplasmic expression and site‐directed mutagenesis 23, and they were characterized by approximately the same expression level under similar cultivation conditions.

**Table 2:** Characteristics of recombinant TEM‐type β‐lactamases

Natural analog of β‐lactamase bearing the mutations studied	Phenotype	Amino acid replacement in recombinant mutant of β‐lactamase TEM‐1		*K*_M_ towards CENTA (μm)	*k*_cat_ (s^−1^)	*k*_cat_/*K*_M_ (μm ^−1^·s^−1^)
		Secondary mutation	Key mutation			
TEM‐1 (wild‐type)	*2b*	—	—	29 ± 3	110 ± 11	3.8
TEM‐2	*2b*	Q39K		26 ± 3	56 ± 7	2.1
TEM‐34	*2br*		M69V	460 ± 40	54 ± 4	0.1
TEM‐135	*2b*	M182T		28 ± 3	75 ± 9	2.7
TEM‐17	*2be*		E104K	12 ± 1	53 ± 7	4.4
TEM‐12	*2be*		R164S	46 ± 4	47 ± 5	1.0
TEM‐160	*2br*	Q39K	M69V	390 ± 35	57 ± 7	0.1
TEM‐18	*2be*	Q39K	E104K	6 ± 1	25 ± 5	4.2
TEM‐7	*2be*	Q39K	R164S	28 ± 3	9 ± 1	0.3
TEM‐129	*2be*	Q39K	R164S and E104K	28 ± 3	20 ± 3	0.7

Table 2 summarizes kinetic data (apparent Michaelis constants (*K*_M_), *k*_cat_ and catalytic efficiency *k*_cat_/*K*_M_) for recombinant enzymes determined against the synthetic chromogenic substrate CENTA based on the antibiotic cephalothin 24. It forms a well‐colored hydrolysis product with a high extinction coefficient. It is noteworthy that CENTA is able to bind effectively with β‐lactamases of different phenotypes, compared with penicillins and cephalosporins, which was reported earlier 27, 28, 29. More effective binding of CENTA together with a higher extinction coefficient for its product allows the study of the subtle effects of mutual influence of mutations in β‐lactamases.

The values of *K*_M_ obtained for the enzymes TEM‐1 and its mutants TEM‐2 and TEM‐135, bearing the single substitutions Q39K and M182T, respectively, were practically identical within the experimental error. These data are consistent with the results obtained earlier for β‐lactamase TEM‐1 and its natural mutant TEM‐2, having the single substitution Q39K 30, 31. This indicates that replacements of glutamine with lysine at position 39 and methionine with threonine at position 182 do not affect significantly the conformation of the active site of the enzyme. At the same time the *K*_M_ values for the mutants having the substitutions at the key positions differ from those for wild‐type TEM‐1 enzyme: the M69V and R164S replacements led to an increase in *K*_M_ by 15 and 1.5 times, respectively; replacement E104K led, on the contrary, to the value of *K*_M_ decreasing by a factor of 2.

The catalytic constant was decreased by 4‐fold for the combination of Q39K and R164S and by 2‐fold for the combination of Q39K and E104K. In first case, catalytic efficiency was also reduced by 3‐fold.

The combination of the secondary mutation Q39K and one of the key mutations shows a decrease in both *K*_M_ and catalytic constant values, which means improved binding of the substrate and less effective catalysis, compared with a single key replacement alone.

### Study of thermal inactivation of mutant forms of TEM‐type β‐lactamases

**Figure 3:**
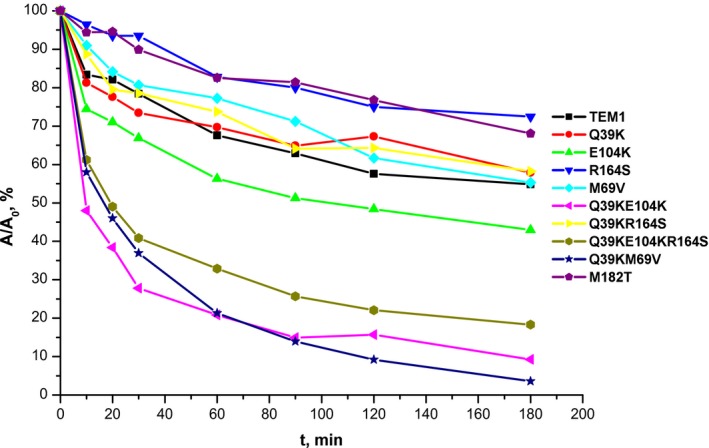
Kinetic curves of residual activity of TEM‐type β‐lactamases with incubation time at 60 °C. Mutant forms of β‐lactamases studied are listed on the right. Enzyme concentration was 1 μm in 50 mm sodium phosphate buffer, pH 7.0.

β‐Lactamases are quite stable enzymes, so to study their ability to show reverse renaturation, we used exposure at 60 °C as a stressful condition for the enzymes. All recombinant forms of TEM‐type β‐lactamases produced in this study were held at elevated temperature for a certain time interval, and then probes were cooled to room temperature and their residual catalytic activity towards the substrate CENTA was measured. The curves of the residual activities against the incubation time at 60 °C are shown in Fig. 3. The curves were not linearized in semi‐logarithmic coordinates, which indicates a complicated multi‐step process of thermal inactivation. This fact correlates with data previously obtained on the refolding of β‐lactamase TEM‐1 in the presence of guanidine hydrochloride 32.

The magnitude of the residual enzymatic activity reflects the ability of enzymes to show reverse renaturation and proper protein refolding. To quantify the effect, we determined the residual activity of the enzymes after exposure at elevated temperature for 3 h (Table 3). The types of effects revealed were divided into three groups: stabilizing (R164S and M182T), destabilizing (E104K, Q39K+E104K, Q39K+EM69V and Q39K+E104K+R164S) and lack of influence (TEM‐1, M69V, Q39K and Q39K+R164S).

**Table 3:** Thermal inactivation of recombinant TEM‐type β‐lactamases. For residual activity, 100% activity corresponds to initial enzymatic activity before exposure to enhanced temperature

Natural analog of β‐lactamase, bearing the mutations studied	Amino acid replacement in recombinant mutant of β‐lactamase TEM‐1	Residual activity of recombinant enzyme after incubation at 60 °C for 3 h (%)
TEM‐1 (wild‐type)	—	58 ± 5
TEM‐2	Q39K	62 ± 5
TEM‐34	M69V	62 ± 5
TEM‐135	M182T	68 ± 7
TEM‐17	E104K	48 ± 5
TEM‐12	R164S	75 ± 9
TEM‐160	Q39K+M69V	12 ± 2
TEM‐18	Q39K+E104K	16 ± 2
TEM‐7	Q39K+R164S	64 ± 7
TEM‐129	Q39K+R164S+E104K	22 ± 4

The single substitutions Q39K and M69V do not affect the rate of thermal inactivation. The single replacement M182T reduces the rate of thermal inactivation, thus stabilizing the enzyme, which corresponds to the literature data on the stabilizing effect of this mutation established by other methods 16, 17. It is worth noting that the natural β‐lactamase TEM‐135, having the mutation M182T, is considered to be one of the most stable variants among all TEM‐type β‐lactamases.

The single replacement E104K, on the contrary, leads to an increase in the inactivation rate. A similar effect is also revealed for double mutants bearing combinations of Q39K with the key mutations M69V and E104K. The destabilizing effect of this secondary mutation in combination with the key mutations is shown for the first time. Unexpectedly, the replacement of R164S in all cases has a pronounced stabilizing effect either in combination with mutation Q39K or in the triple mutant together with Q39K and E104K, reducing the rate of thermal inactivation.

### Structural analysis of β‐lactamase TEM‐1 and its mutants bearing Q39K substitution

**Figure 4:**
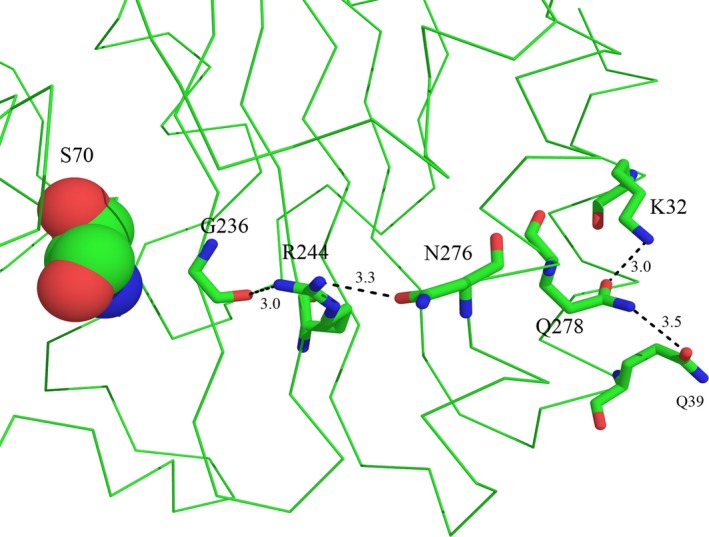
Hydrogen bonds network from Q39 to G236 in β‐lactamase TEM‐1 (PDB code 1BTL). S70 is shown in CPK form, and the side chains of the rest of the residues are shown as sticks. Dashed lines show hydrogen bonds.

To explain the experimental data obtained, a computer analysis of β‐lactamase TEM‐1 and its mutant forms was carried out on the basis of available crystal structures. In the spatial structure of β‐lactamase TEM‐1 (PDB code 1BTL), residue Q39 is located on the protein surface near the C end of the first α‐helix and is separated from the enzyme's catalytic site by a β‐sheet (Fig. 2). We focused on finding possible links between residue Q39 and the active center residues around S70. The side chain conformation of Q39 and solved water molecule in close proximity provide strong evidence of this amino acid being solvated, which serves as a protein stabilizing factor. There is one crystallographic water molecule in the structure with a distance between its oxygen and the Q39 nitrogen of 2.8 Å, and this indicates that a hydrogen bond exists between these two atoms. The side chain oxygen OE1 of Q39 and NE2 of Q278 are 3.5 Å apart, which is enough to form a hydrogen bond (the second one for Q39). The OE1 atom of Q278 might establish a hydrogen bond with the NH_3_ group of K32 (the distance is 2.96 Å). Residue Q278 belongs to the C‐terminal α‐helix of the protein and is preceded by N276, whose side chain is directed towards the β‐sheet of the enzyme. The OD1 atom of N276 establishes a hydrogen bond with the NH_2_ group of R244 (the distance is 3.25 Å). This hydrogen bond enforces the interaction between the elements of the protein's secondary structure, enhancing its thermal stability. ND2 of N276 serves as a hydrogen donor with a bound crystallographic water molecule (the distance is 2.92 Å). Solvation of N276 also increases the thermal stability of the protein. A second nitrogen atom, NH1 of R244, forms a hydrogen bond with the main chain carbonyl oxygen of G236 (the distance is 3.03 Å), providing an extra bond between the strands of enzyme β‐sheet. Figure 4 shows the network of hydrogen bonds revealed from residues Q39 and K32 to residue G236 through the residues Q278, N276 and R244. Residues G236 and R244 are located in the vicinity of the catalytic site. So, one can say that Q39 and K32 are linked to the enzyme's active site by a hydrogen bond network, which enforces the links between the protein's secondary structure elements (N‐ and C‐terminal protein helices, strands of β‐sheet) and stabilizes the protein.

To simulate changes in the β‐lactamase molecule upon replacement of glutamine at position 39 with lysine, we looked for available β‐lactamase structures having this substitution.There is only one solved structure of β‐lactamase TEM‐72 (PDB code 3P98) bearing a mutation Q39K in combination with the key mutations G238S and E240K and secondary mutation M182T 25. It was used for comparative analysis, in which we followed the numbering of the residues in accordance with the Ambler scheme 33. It starts from His26, has Ser70 as a catalytic residue and two frame shifts in the area of 236–241 (without 239) and 251–256 (without 253). The numbering in 3P98 is consecutive and did not take into account one of the frame shifts. Therefore, in our analysis there is a discrepancy in the numbering, and residues N276/Q728 correspond to N275/Q277 in 3P98.

**Figure 5:**
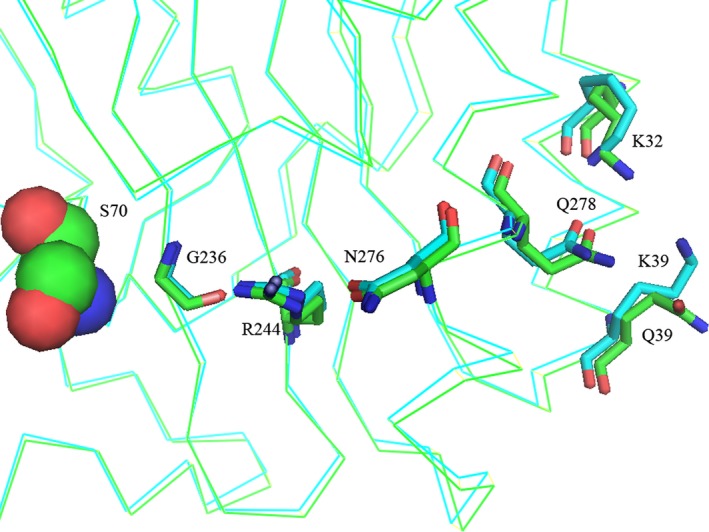
Hydrogen bond network from residue 39 to catalytic sites of β‐lactamases TEM‐1 (PDB code 1BTL) and TEM‐72 (PDB code 3P98). S70 is shown in CPK form. Polypeptide chain folding and side chain carbons of β‐lactamase TEM‐1 are shown in green, and those of β‐lactamase TEM‐72 are shown in blue.

The structures of β‐lactamases TEM‐1 and TEM‐72 look very similar. The root mean square deviation (RMSD) between the polypeptide chain heavy atoms of these structures is only 0.7 Å, which comes mainly from the difference in folding of the loop formed by residues 252–257. In the rest, the two proteins folds are very similar, with RMSD around 0.1 Å. Figure 5 shows the structures 1BTL and 3P98 superimposed with the network of hydrogen bonds revealed for the structure 1BTL. One can expect that mutation Q39K might cause the loss of one hydrogen bond in the chain, decreasing the protein's stability. Because of the positive charge of the terminal nitrogen group, lysine is not able to establish the hydrogen bond with the nitrogen of Q278. As a result, the Q278 side chain is shifted towards K32 creating an enforced hydrogen bond between the oxygen of Q278 and the nitrogen of K32 (the distance between these two atoms is 2.9 Å, which is less than between the corresponding atoms in the structure of β‐lactamase TEM‐1). There are no bound water molecules around the residue K39 in β‐lactamase TEM‐72, which may indicate that it is less solvated. The rest of the residues forming the hydrogen bond network look very similar as regards side chain conformation and the distances between them for both TEM‐1 and TEM‐72 enzymes.

The search for conformational changes that accompany the Q39K mutation did not reveal any substantial changes in neighboring residues. One can expect that this mutation will not affect the conformation of the residues forming the enzyme's catalytic site. So the binding affinity of the mutated enzyme for substrate should remain the same as for the wild‐type. This result is in agreement with approximately the same values of *K*_M_ for β‐lactamase TEM‐1 and TEM‐2 with the Q39 substitution.

**Figure 6:**
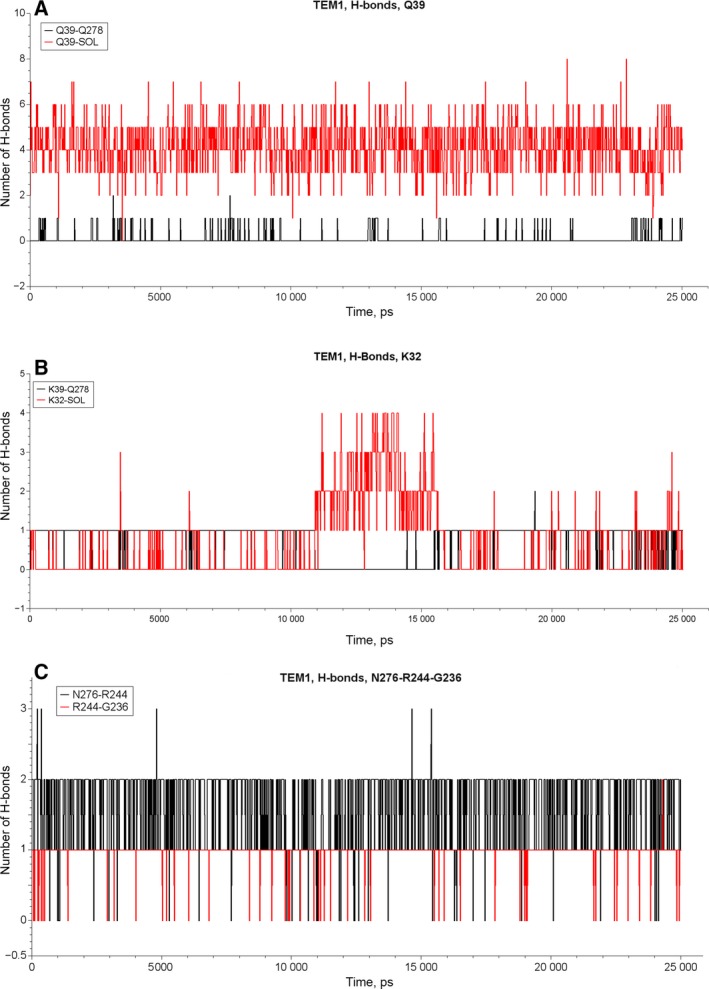
The number of hydrogen bonds in the course of MD simulations of β‐lactamase TEM‐1 for the pairs Q39–Q278 and Q39–solvent water molecules (A), for the pairs K32–Q278 and K32–solvent water molecules (B), and for the pairs N276–R244 and R244–G236 (C).

**Figure 7:**
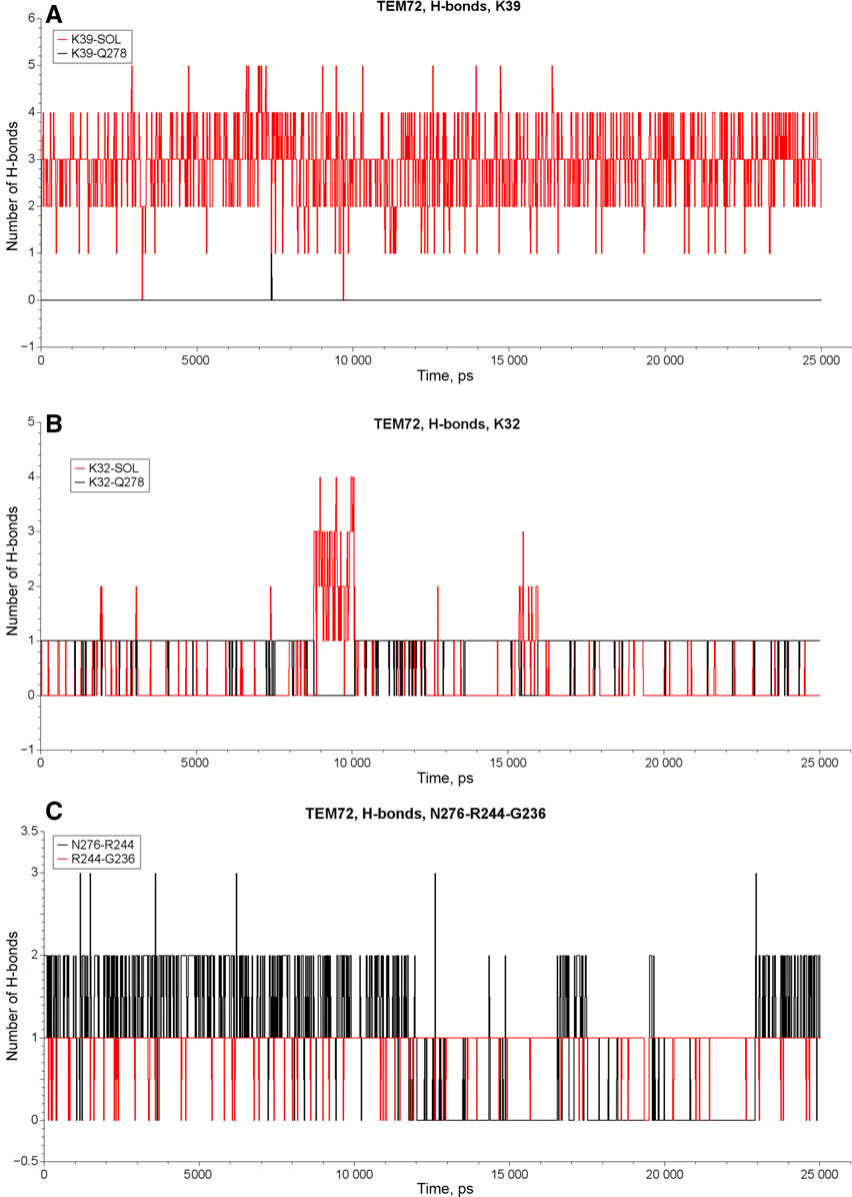
The number of hydrogen bonds in the course of MD simulations of β‐lactamase TEM‐72 for the pairs Q39–Q278 and Q39–solvent water molecules (A), for the pairs K32–Q278 and K32–solvent water molecules (B), and for the pairs N276–R244 and R244–G236 (C).

In order to estimate the mobility and degree of solvation of the residues involved in the hydrogen bond network we performed MD simulations for β‐lactamases TEM‐1 and TEM‐72. The MD trajectories obtained for each protein were processed to calculate the number of established hydrogen bonds in frames separated from each other by 200 ps. Dynamic stability of hydrogen bonds was analyzed for residue pairs Q(K)39–Q278, K32–Q278, N276–R244 and R244–G236. We used the criterion of maximal distance between the acceptor and the donor of the hydrogen bond of 3 Å to consider the bond as established. The results of the simulations are shown in Figs 6 and 7. Table 4 presents data analysis on estimation of lifetime for selected residue pairs of β‐lactamases TEM‐1 and TEM‐72. Hydrogen bond Q39–Q278 in β‐lactamase TEM‐1 was presented only in 10% of analyzed conformations, i.e. during 10% of MD trajectories. The rest of the time residue Q39 was completely solvated, establishing extra hydrogen bonds with water molecules. The residue K39 in β‐lactamase TEM‐72 never formed a hydrogen bond with Q278 and remained completely solvated. Hydrogen bond K32–Q278 was observed for 75% of TEM‐1 trajectories and for 88% of TEM‐72 trajectories. Thus, it was quite stable in both enzymes. Nevertheless, mutation Q39K resulted in a redistribution of interaction of residue Q278. The disappearance of its hydrogen bond with K39 in β‐lactamase TEM‐72 was compensated by the strengthening of the hydrogen bond in the pair K32–Q278 and the reduction of the hydrogen bond in the pair N276–R244, which became unsustainable and its lifetime was halved compared with TEM‐1. The hydrogen bond R244–G236 had the same stability (about 95% of trajectory time) for both β‐lactamases TEM‐1 and TEM‐72.

**Table 4:** Percentage of time during MD simulations when hydrogen bonds were formed between selected residues of β‐lactamases TEM‐1 and TEM‐72

Residue pairs	Lifetime of hydrogen bond (%)	
	TEM‐1	TEM‐72
Q39–Q278	10	—
K39–Q278	—	0
K32–Q278	75	88
N276–R244	98	45
R244–G236	96	95

## Discussion

β‐Lactamases continue to be an attractive model for investigating the mutual influence of mutations due to their evolutionary variability. The term epistasis is increasingly used to explain polymorphism of β‐lactamases in the context of their molecular evolution and adaptation, although the molecular basis of the phenomenon still remains obscure 34, 35, 36. Non‐linear and non‐additive effects (beneficial or deleterious) of epistatic mutations were found to influence the protein fitness landscape and limit the evolvability 37, 38. Surveys of β‐lactamase polymorphism, in particular for TEM‐type enzymes, made it possible to divide single amino acid substitutions into two distinct groups: so called key and secondary or associated mutations. While the former, changing the architecture of the enzyme's active site, provide new properties, namely, expansion of substrate specificity and facilitation of inhibitor resistance, the latter are located far from the active center and their role is poorly understood. The hypothesis is currently accepted that such mutations compensate the fitness defects introduced by key mutations, representing an activity–stability tradeoff. Earlier this was confirmed for the M182T substitution, present in ESBL and IR TEM β‐lactamases, which is thought to act as a suppressor of protein rigidity loss 16, 17.

Here we have attempted to shed light on the possible role of the secondary Q39K mutation in TEM‐type β‐lactamases. It attracted our attention as it is located on the surface of a protein globule and is quite frequently observed in different β‐lactamase phenotypes both as a single mutation and in combination with the key substitutions as well. We confirmed the data earlier obtained that a single replacement of Q39K in β‐lactamase TEM‐2 does not lead to any appreciable changes of the enzyme's properties, which is in agreement with the remoteness of this substitution from the enzyme's active site 30. Kinetic data obtained showed various influence of single replacements on catalytic properties and thermal stability of corresponding β‐lactamase mutants compared with TEM‐1 enzyme (Tables 2 and 3). But for the combination of Q39K with the key substitutions related to both ESBL (E104K, R164S) and IR (M69V) phenotypes, new properties were found: the *K*_M_ values in all cases were decreased, the catalytic constants were decreased for ESBL phenotypes, and the thermal stability of mutant forms containing Q39K combinations was also reduced.

Studying the thermal stability of different TEM‐1 β‐lactamase mutants, we suggested not limiting the melting temperature (*T*_m_) estimation and to analyze the influence of mutations on the fitness of a protein globule. It is important to study the dynamic stability of the structure, which is manifest in the ability of the enzyme to restore the catalytically active conformation after removal of the denaturing pressure. Considering this, the destabilizing effect of Q39K combinations with the key mutations means a reduction of the enzyme's ability to reverse thermal reactivation, which is especially pronounced in combination with the E104K and M69V substitutions. Our data demonstrate a non‐additive negative effect of these mutation combinations, which is partially in accordance with the unfavorable influence of the number of positively charged residues on stability of β‐lactamases 39.

Structural analysis of Q39K mutation in combination with other mutations in TEM‐type β‐lactamases revealed a hydrogen bond network from the surface‐located residues Q39 and K32 to the residues R244 and G236 located in the vicinity of the active site. MD simulations have revealed the changes in hydrogen bond lifetime. The most pronounced effect was noted for the conformation and mobility of residue N276. As a result, the strength of the N276–R244 hydrogen bond decreases, making this region of protein globule less rigid. In addition, it can be noted that any replacement of R244 by an amino acid that is not a hydrogen bond donor will lead to the loss of the hydrogen bond between the residues R244 and G236. This brings structural distortion in close proximity to the enzyme's catalytic site and might have an influence on activity and thermal stability of the protein.

Mutant forms of β‐lactamase having the key substitution R164S manifested a decrease in thermal inactivation rate in all combinations with other replacements compared with those with R164 intact (Table 3). Moreover, the inactivation curve for the R164S mutant (Fig. 3) was similar to that of the most stable β‐lactamase, TEM‐135 with the M182T substitution 16, 17. Residue 164 is located in the so called Ω‐loop (residues 164–179), which forms the bottom part of the active site and represents the conservative structural element among all β‐lactamases of molecular class A. Ω‐Loop conformation is crucial for proper orientation of the catalytically active glutamate residue (E166), which participates in the deacylation step of β‐lactam hydrolysis together with N170 7. An arginine residue (R164) forms ionic bonds with E171 and D179, and thus substitution R164S results in an increased flexibility of the Ω‐loop and enhanced ability to hydrolyze the extended‐spectrum cephalosporins. Indeed, the results of the crystallographic study indicate an increased conformational flexibility of the Ω‐loop in the case of the R164S mutation 34.

Thermodynamic stability of different β‐lactamases was previously investigated and interpreted in terms of *T*_m_ of the protein globule 16. In particular, *T*_m_ for β‐lactamase TEM‐12 with the R164S substitution was reported to be lower by 8.8 °C in comparison with that for the most stable β‐lactamase, TEM‐135 with the M182T substitution. Our data on the ability of certain β‐lactamases to show reverse thermal reactivation do not correlate with these *T*_m_ values, which refer largely to fitness and rigidity of the protein globule itself. An apparent contradiction can be explained within the framework of the β‐lactamase refolding theory 32, 40, 41. As was shown earlier, the refolding process of β‐lactamase TEM‐1 consists of at least five stages, which in turn split into an initial fast (3–50 ms) and a final slow (>300 ms) phase 32. The last stage of enzyme refolding is supposed to be associated with *cis–trans* isomerization of the peptidyl–proline bond (E166–P167). Only the *cis*‐isomer demonstrates catalytic activity, providing proper orientation of the catalytically important glutamate in position 166. Based on the data obtained, we may suggest that all studied amino acid substitutions might affect the isomerization of this bond, which in turn leads to a different ratio of *cis*‐/*trans*‐isomers and therefore to active or inactive conformations of the enzyme. The antitropic effect of the Q39K and R164S substitutions may apparently be associated with an increase/decrease in the energy barrier of the *cis–trans* transition, which is about 20 kcal·mol^−1^ 42.

Currently, the spread of antibiotic resistance is certainly connected with anthropogenic factors. However, the phenomenon of bacterial resistance has deeper roots and is much older than the era of the use of antibiotics by humans. Broad polymorphism in the superfamily of β‐lactamases and the opposite effects of certain mutations on kinetic properties and stability appear to play a significant role in the adaptation and survival of microorganisms in changing external conditions. The literature data indicate that both mutation types, stabilizing protein structure and contrary ones, arise in the course of evolutionary adaptation of bacteria. Our study underlines the relevance of a systematic comprehensive study of the effect of so‐called secondary or ‘silent’ mutations in combination with the key ones on the properties of β‐lactamases, which helps in understanding the evolution of β‐lactamases. The study of the surface spots on the protein globule, where the residues affecting protein susceptibility to external influence are located, facilitates the search for new targets for directed β‐lactamase inhibition and overcoming resistance.

## Author contributions

AE supervised the project; MR, VG and AE designed the research; VG, IA, OS, MU and II performed the research; IU, AV and DS performed modeling and molecular dynamics; VG, MR, IU analyzed data; MR, VG, IU, AV and AE wrote the paper with contributions from the other authors.

## References

[1] Bush K and Bradford PA (2016) β‐Lactams and β‐lactamase inhibitors: An overview. Cold Spring Harb Perspect Med 6, 1–22. a025247 10.1101/cshperspect.a025247 PMC4968164 27329032

[2] Antimicrobial Resistance Global Report on surveillance . (2014) World Health Organization http://www.who.int/drugresistance/en/.

[3] Tang S , Apisarnthanarak A and Hsu LY (2014) Mechanisms of β‐lactam antimicrobial resistance and epidemiology of major community‐and healthcare‐associated multidrug‐resistant bacteria. Adv Drug Delivery Rev 78, 3–13. 10.1016/j.addr.2014.08.003 25134490

[4] Baquero F , Lanza VF , Canton R and Coque TM (2015) Public health evolutionary biology of antimicrobial resistance: priorities for intervention. Evol Appl 8, 223–239. 25861381 10.1111/eva.12235 PMC4380917

[5] Pitout JD and Laupland KB (2008) Extended‐spectrum β‐lactamase‐producing *Enterobacteriaceae*: an emerging public‐health concern. Lancet Infect Dis 8, 159–166. 18291338 10.1016/S1473-3099(08)70041-0

[6] Bush K and Jacoby GA (2010) Updated functional classification of β‐lactamases. Antimicrob Agents Chemother 54, 969–976. 19995920 10.1128/AAC.01009-09 PMC2825993

[7] Pimenta AC , Fernandes R and Moreira IS (2014) Evolution of drug resistance: insight on TEM β‐lactamases structure and activity and β‐lactam antibiotics. Mini Rev Med Chem 14, 111–122. 24456272 10.2174/1389557514666140123145809

[8] Salverda MLM , Dellus E , Gorter FA , Debets AJM , van der Oost J , Hoekstra RF , Tawfik DS and de Visser JA (2011) Initial mutations direct alternative pathways of protein evolution. PLoS Genet 7, e1001321. 21408208 10.1371/journal.pgen.1001321 PMC3048372

[9] Partridge SR and Hall RM (2005) Evolution of transposons containing blaTEM genes. Antimicrob Agents Chemother 49, 1267–1268. 15728947 10.1128/AAC.49.3.1267-1268.2005 PMC549287

[10] García‐Cobos S , Arroyo M , Campos J , Pérez‐Vázquez M , Aracil B , Cercenado E , Orden B , Lara N and Oteo J (2013) Novel mechanisms of resistance to β‐lactam antibiotics in *Haemophilus parainfluenzae*: β‐lactamase‐negative ampicillin resistance and inhibitor‐resistant TEM β‐lactamases. J Antimicrob Chemother 68, 1054–1059. 23335113 10.1093/jac/dks525

[11] Ríos E , López MC , Rodríguez‐Avial I , Pena I and Picazo JJ (2015) Characterization of inhibitor‐resistant TEM β‐lactamases and mechanisms of fluoroquinolone resistance in *Escherichia coli* isolates. Microb Drug Resist 21, 512–515. 25945693 10.1089/mdr.2015.0039

[12] Baraniak A , Fiett J , Mrowka A , Walory J , Hryniewicz W and Gniadkowski M (2005) Evolution of TEM‐type extended‐spectrum β‐lactamases in clinical *Enterobacteriaceae* strains in Poland. Antimicrob Agents Chemother 49, 1872–1880. 15855509 10.1128/AAC.49.5.1872-1880.2005 PMC1087658

[13] Abriata LA , Salverda ML and Tomatis PE (2012) Sequence‐function‐stability relationships in proteins from datasets of functionally annotated variants: the case of TEM β‐lactamases. FEBS Lett 586, 3330–3335. 22850115 10.1016/j.febslet.2012.07.010

[14] Zeil C , Widmann M , Fademrecht S , Vogel C and Pleiss J (2016) Network analysis of sequence‐function relationships and exploration of sequence space of TEM β‐lactamases. Antimicrob Agents Chemother 60, 2709–2717. 26883706 10.1128/AAC.02930-15 PMC4862526

[15] Guthrie VB , Allen J , Camps M and Karchin R (2011) Network models of TEM β‐lactamase mutations coevolving under antibiotic selection show modular structure and anticipate evolutionary trajectories. PLoS Comput Biol 7, e1002184. 21966264 10.1371/journal.pcbi.1002184 PMC3178621

[16] Wang X , Minasov G and Shoichet BK (2002) Evolution of an antibiotic resistance enzyme constrained by stability and activity trade‐offs. J Mol Biol 320, 85–95. 12079336 10.1016/S0022-2836(02)00400-X

[17] Brown NG , Pennington JM , Huang W , Ayvaz T and Palzkill T (2010) Multiple global suppressors of protein stability defects facilitate the evolution of extended‐spectrum TEM β‐lactamases. J Mol Biol 404, 832–846. 20955714 10.1016/j.jmb.2010.10.008 PMC3032993

[18] Shcherbinin DS , Rubtsova MYU , Grigorenko VG , Uporov IV , Veselovsky AV and Egorov AM (2017) Investigation of the role of mutations M182T and Q39K in structure of β‐lactamase TEM‐72 by molecular dynamics method. Biochemistry (Moscow) Suppl Series B 11, 120–127.

[19] Du Bois SK , Marriott MS and Amyes SG (1995) TEM‐ and SHV‐derived extended‐spectrum β‐lactamases: relationship between selection, structure and function. J Antimicrob Chemother 35, 7–22. 7768784 10.1093/jac/35.1.7

[20] Laemmli UK (1970) Cleavage of structural proteins during the assembly of the head of bacteriophage T4. Nature 227, 680–685. 5432063 10.1038/227680a0

[21] Sambrook J , Fritsch EF and Maniatis T (1989) Molecular Cloning: A Laboratory Manual, 2nd edn. Cold Spring Harbor Laboratory Press, Cold Spring Harbor, NY.

[22] Braman J , Papworth C and Greener A (1996) Site‐directed mutagenesis using double‐stranded plasmid DNA templates. Methods Mol Biol 57, 31–44. 8849992 10.1385/0-89603-332-5:31

[23] Grigorenko VG , Andreeva IP , Rubtsova MYU , Deygen IM , Antipin RL , Majouga AG , Egorov AM , Beshnova DA , Kallio J , Hackenberg C *et al* (2017) Novel non‐β‐lactam inhibitor of β‐lactamase TEM‐171 based on acylated phenoxyaniline. Biochimie 132, 45–53. 27771370 10.1016/j.biochi.2016.10.011

[24] Bebrone C , Moali C , Mahy F , Rival S , Docquier JD , Rossolini GM , Fastrez J , Pratt RF , Frère JM and Galleni M (2001) CENTA as a chromogenic substrate for studying β‐lactamases. Antimicrob Agents Chemother 45, 1868–1871. 11353639 10.1128/AAC.45.6.1868-1871.2001 PMC90559

[25] Docquier JD , Benvenuti M , Calderone V , Rossolini GM and Mangani S (2011) Structure of the extended‐spectrum β‐lactamase TEM‐72 inhibited by citrate. Acta Crystallogr Sect F Struct Biol Cryst Commun 67, 303–306. 10.1107/S1744309110054680 PMC3053151 21393831

[26] Jelsch C , Mourey L , Masson JM and Samama JP (1993) Crystal structure of *Escherichia coli* TEM‐1 β‐lactamase at 1.8 A resolution. Proteins 16, 364–383. 8356032 10.1002/prot.340160406

[27] Robin F , Delmas J , Archambaud M , Schweitzer C , Chanal C and Bonnet R (2006) CMT‐type β‐lactamase TEM‐125, an emerging problem for extended‐spectrum β‐lactamase detection. Antimicrob Agents Chemother 50, 2403–2408. 16801418 10.1128/AAC.01639-05 PMC1489774

[28] Neuwirth C , Labia R , Siebor E , Pechinot A , Madec S , Chaibi EB and Kazmierczak A (2000) Characterization of TEM‐56, a novel β‐lactamase produced by a *Klebsiella pneumoniae* clinical isolate. Antimicrob Agents Chemother 44, 453–455. 10639384 10.1128/aac.44.2.453-455.2000 PMC89705

[29] Raquet X , Lamotte‐Brasseur J , Fonze E , Gousard S , Courvalin P and Frere JM (1994) TEM β‐lactamase mutants hydrolysing third‐generation cephalosporins. A kinetic and molecular modelling analysis. J Mol Biol 244, 625–639. 7990143 10.1006/jmbi.1994.1756

[30] Blazquez J , Morosini MI , Negri MC , Gonzalez‐Leiza M and Baquero F (1995) Single amino acid replacements at positions altered in naturally occurring extended‐spectrum TEM β‐lactamases. Antimicrob Agents Chemother 39, 145–149. 7695296 10.1128/aac.39.1.145 PMC162500

[31] Bermudes H , Jude F , Chaibi EB , Arpin C , Bebear C , Labia R and Quentin C (1999) Molecular characterization of TEM‐59 (IRT‐17), a novel inhibitor‐resistant TEM‐derived β‐lactamase in a clinical isolate of *Klebsiella oxytoca* . Antimicrob Agents Chemother 43, 1657–1661. 10390218 10.1128/aac.43.7.1657 PMC89339

[32] Vanhove M , Lejeune A and Pain RH (1998) β‐Lactamases as models for protein‐folding studies. Cell Mol Life Sci 54, 372–377. 9614975 10.1007/s000180050166 PMC11147380

[33] Ambler RP (1991) A standard numbering scheme for the class A beta‐lactamases. Biochem J 276, 269–270. 2039479 10.1042/bj2760269 PMC1151176

[34] Dellus‐Gur E , Elias M , Caselli E , Prati F , Salverda ML , de Visser JA , Fraser JS and Tawfik DS (2015) Negative epistasis and evolvability in TEM‐1 β‐lactamase ‐ the thin line between an enzyme's conformational freedom and disorder. J Mol Biol 427, 2396–2409. 26004540 10.1016/j.jmb.2015.05.011 PMC4718737

[35] Breen MS , Kemena C , Vlasov PK , Notredame C and Kondrashov FA (2012) Epistasis as the primary factor in molecular evolution. Nature 490, 535–538. 23064225 10.1038/nature11510

[36] Orencia MC , Yoon JS , Ness JE , Stemmer WP and Stevens RC (2001) Predicting the emergence of antibiotic resistance by directed evolution and structural analysis. Nat Struct Biol 8, 238–242. 11224569 10.1038/84981

[37] De Visser JA and Krug J (2014) Empirical fitness landscapes and the predictability of evolution. Nat Rev Genet 15, 480–490. 24913663 10.1038/nrg3744

[38] Weinreich DM , Delaney NF , Depristo MA and Hartl DL (2006) Darwinian evolution can follow only very few mutational paths to fitter proteins. Science 312, 111–114. 16601193 10.1126/science.1123539

[39] Vanhove M , Houba S , Lamotte‐Brasseur J and Frère JM (1995) Probing the determinants of protein stability: comparison of class A β‐lactamases. Biochem J 308, 859–864. 8948443 10.1042/bj3080859 PMC1136803

[40] Vanhove M , Raquet X , Palzkill T , Pain RH and Frère JM (1996) The rate‐limiting step in the folding of the cis‐Pro167Thr mutant of TEM‐1 β‐lactamase is the trans to cis isomerization of a non‐proline peptide bond. Proteins 25, 104–111. 8727322 10.1002/(SICI)1097-0134(199605)25:1<104::AID-PROT8>3.0.CO;2-J

[41] Vanhove M , Raquet X and Frère JM (1995) Investigation of the folding pathway of the TEM‐1 β‐lactamase. Proteins 22, 110–118. 7567959 10.1002/prot.340220204

[42] Andreotti AH (2003) Native state proline isomerization: an intrinsic molecular switch. Biochemistry 42, 9515–9524. 12911293 10.1021/bi0350710

